# How to accurately assess surfactant biodegradation-impact of sorption on the validity of results

**DOI:** 10.1007/s00253-019-10202-9

**Published:** 2019-11-15

**Authors:** Dorota Cierniak, Marta Woźniak-Karczewska, Anna Parus, Bogdan Wyrwas, Andreas P. Loibner, Hermann J. Heipieper, Łukasz Ławniczak, Łukasz Chrzanowski

**Affiliations:** 1 Institute of Chemistry and Technical Electrochemistry, Poznan University of Technology, Bedrychowo 4, 60-965 Poznan, Poland; 2 Institute of Chemical Technology and Engineering, Poznan University of Technology, Pl. M. Skłodowskiej-Curie 2, 60-965 Poznan, Poland; 3 IFA-Tulln, BOKU-University of Natural Resources and Life Sciences, 3430 Vienna, Tulln Austria; 4 Department of Environmental Biotechnology, Helmholtz Centre for Environmental Research – UFZ, Permoserstraße 15, 04318 Leipzig, Germany

**Keywords:** Activated sludge, *Bacillus cereus*, Biodegradation, *Pseudomonas putida*, *Saccharomyces cerevisiae*, Sorption, Surfactant

## Abstract

Surfactants not only are widely used in biotechnological processes but also constitute significant contaminants of the modern world. Among many reports, there is a shortage of works which summarize the issue of surfactant sorption to biomass in a way that would elucidate the biological factors for analysts and analytical factors for microbiologists. The main factor, which is not as obvious as one would expect, is associated with the susceptibility of analytical approaches to errors resulting from incorrect handling of biomass. In case of several publications reviewed in the framework of this study, it was not possible to establish whether the decrease of the analytical signal observed by the authors actually resulted from biodegradation of the surfactant. This review emphasizes the necessity to consider the possibility of surfactant sorption to microbial cells, which may result in significant detection errors as well as conceptual inconsistency. In addition, a reference study regarding representative surfactants (cationic, anionic and non-ionic) as well as yeast, Gram-negative, Gram-positive bacteria, and activated sludge was provided to highlight the possible errors which may arise from disregarding sorption processes when determining degradation of surfactants. This particularly applies to systems which include ionic surfactants and activated sludge as sorption may account for 90% of the observed depletion of the surfactant. Therefore, a systematic approach was proposed in order to improve the credibility of the obtained results. Finally, the need to employ additional procedures was highlighted which may be required in order to verify that the decrease of surfactant concentration results from biodegradation processes.

**Electronic supplementary material:**

The online version of this article (10.1007/s00253-019-10202-9) contains supplementary material, which is available to authorized users.

## Introduction

Surfactants, or surface active agents, comprise a structurally diverse group of chemical compounds which is extensively used in everyday applications. They are present in common cleaning agents, fabric softeners, domestic detergents, and hygienic products such as shampoos, shower gels, and toothpastes (Blagojević et al. 2016; Kurrey et al. 2019). Furthermore, they are commonly utilized as emulsifiers, dispersants, wetting, and foaming agents in several branches of the industry (e.g., in the cosmetic, pharmaceutical, agricultural, food, textile, polymer, and paint sectors) (Traverso-Soto et al. 2016; Palmer and Hatley 2018). Currently, non-ionic surfactants are the dominant group with approx. 40% of the total market share, which experienced a steady increase of demand during the last years (Allied Market Research 2017). Anionic surfactants are the next commonly used on the market because of their numerous applications and cost-efficient production. Cationic surfactants are more expensive and, therefore, constitute a smaller share of the market. In the last decades, surfactants of biological origin, biosurfactants, have gained increasing interest, due to their environmental friendliness (Chrzanowski et al. 2012a; Ławniczak et al. 2013). However, their physicochemical behavior is similar to their synthetic counterparts (Chrzanowski et al. 2012b; Owsianiak et al. 2009a, b).

As a consequence, surfactants are the major constituents of both municipal (Aloui et al. 2009) and industrial wastewater (Ławniczak and Marecik 2019). In addition, they are intentionally introduced into the environment in high amounts for the remediation of oil spills (Peziak et al. 2013; Szulc et al. 2014; Trellu et al. 2016; Lamichhane et al. 2017; Ren et al. 2018)—for example, approximately 7 million liters of Corexit® were applied after the Deepwater Horizon oil spill in 2010 (Rahsepar et al. 2016; McFarlin et al. 2018).

Due to their common discharge into the environment, surfactants are among the most important environmental pollutants (Wyrwas et al. 2013; Menzies et al. 2017). They often exhibit potent biological activity, i.e., interaction with cellular membranes and disruption of important biochemical processes, and may therefore negatively affect living organisms (especially in case of marine environments) (Wyrwas et al. 2011; Rebello et al. 2014; Pereira et al. 2015). As a result, it is imperative to monitor the concentration of surfactants and to keep the amount of surfactants remaining in the environment at minimum by developing efficient methods of their (biological) elimination (Garcia et al. 2016; Sakai et al. 2017; Atashgahi et al. 2018) and replacing more persistent surfactants by biodegradable alternatives (Chrzanowski et al. 2009, 2011). The importance of these tasks is highlighted by several comprehensive reviews regarding the fate of surfactants in the environment (Mungray and Kumar 2009; Könnecker et al. 2011; Cowan-Ellsberry et al. 2014; Jackson et al. 2016). The relevance of this topic is confirmed by the fact that numerous scientific reports focused on the biodegradation of surfactants were published to date (Kara Murdoch et al. 2018; Fedeila et al. 2018; Barra Caracciolo et al. 2019; Nguyen and Oh 2019).

**Fig. 1:**
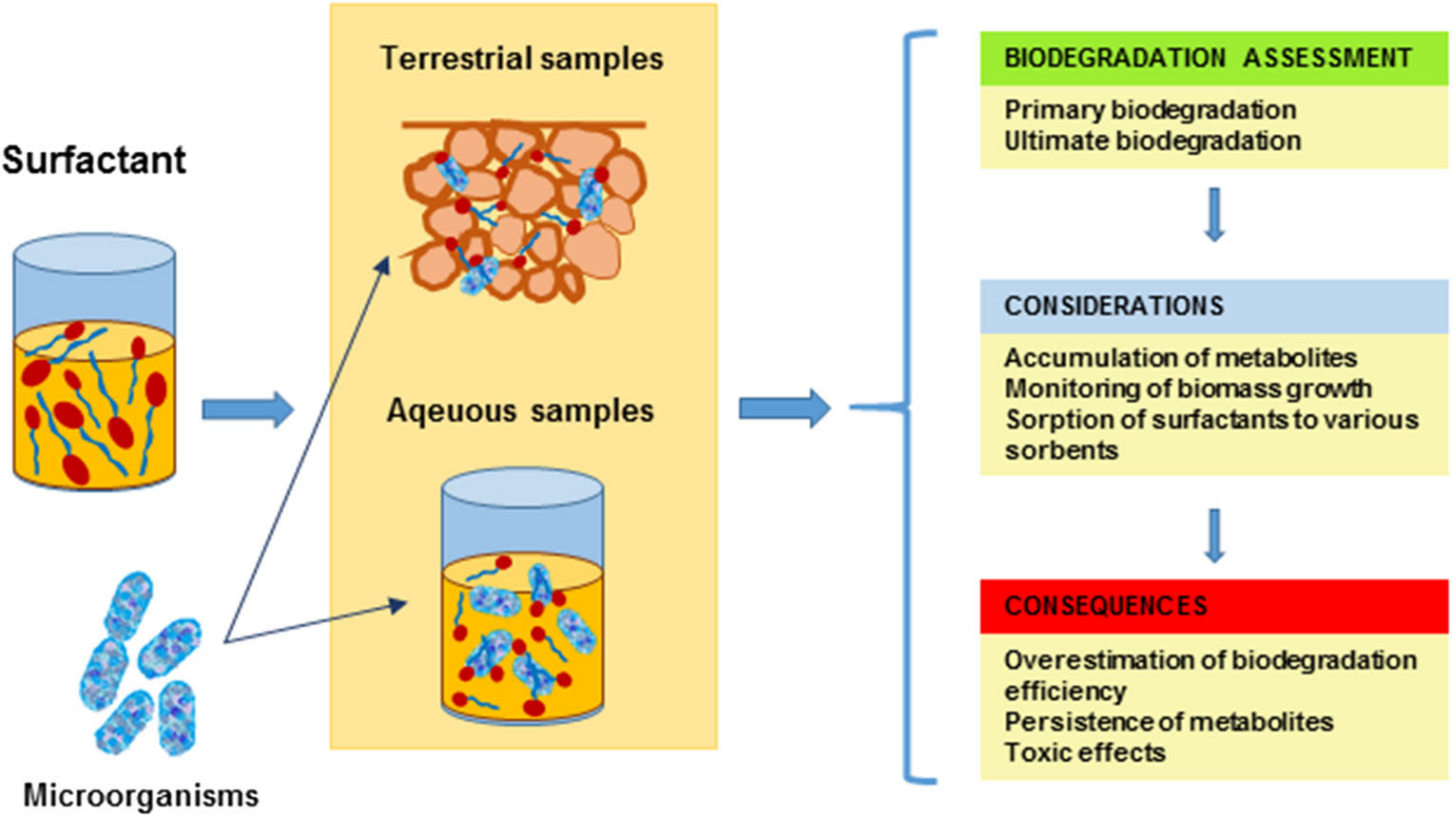
Conceptual summary of considerations and possible consequences which should be considered in surfactant biodegradation studies

However, it should be noted that the protocols used for assessment of surfactant biodegradability differ notably in terms of the quality of obtained results (Fig. 1). Numerous approaches regarding the determination of surfactants are based solely on the decrease of the analytical signal attributed to the concentration of the studied compound (Zembrzuska et al. 2016). While such methods allow for a relatively rapid and simple measurement, the factual value of the produced data may be limited. In these approaches, several crucial considerations, such as the possible biotransformation of the initial surfactant structure into a stable and potentially more toxic metabolite, are not always taken into account. Incomplete biodegradation often results in the formation of more toxic metabolites, which are by far more hazardous than the parent compound, or derivatives with relatively stable structure, which may accumulate in the environment. Both cases result in major environmental risks despite the fact that the biodegradation assay may suggest complete removal of the initial structure. The lack of biomass growth monitoring methods leads to false assumptions, especially in case of systems with multiple carbons sources. Furthermore, surfactants often exhibit a notable tendency for sorption (He et al. 2015). Although this issue is usually addressed in case of environmental matrices, particularly in case of sediment or soil samples (Corada-Fernández et al. 2018), the fact that sorption of surfactants to biomass may also occur is often overlooked. This corresponds well with the case of mycotoxins, which often exhibit a tendency to bind to the cell pellet, especially in case of lactic acid bacteria. Haskard et al. (2001) observed the extracellular binding of aflatoxin-B1 to the surface of *Lactobacillus rhamnosus* and established that this process is based on weak non-covalent interactions. Furthermore, El-Nezami et al. (2002) reported that approx. 55% of zearalenone was instantly bound to the cells of *Lactobacillus* strains and indicated that the binding efficiency is directly influenced by the bacterial concentration. The studies imply that binding of toxins on the surface of cells may often be their major removal mechanism. While this phenomenon is positive in terms of reducing the impact of mycotoxins on human health, it is a major disadvantage in terms of analytical procedures. It should also be emphasized that binding may also occur in case of dead biomass, which makes the quantification of biodegradation processes even more challenging. This may contribute to significant consequences, which include notable errors in terms of actual quantification of surfactants, misinterpretation of data regarding their actual environmental impact, and, in consequence, to inconsistency in the current state of the art.

In the framework of this study, recent reports focusing on surfactant biodegradation were reviewed in terms of variety of the employed analytical approaches and the consistency of obtained data with the established conclusions. Furthermore, the importance of surfactant sorption to biomass is highlighted on the basis of original research results. The aim of this review is to (i) identify the limitations of different methods used for the determination of surfactant biodegradation, to (ii) discuss the issues which may be a common source of scientific bias, and to (iii) elucidate adequate guidelines for future experiments used for the assessment of the environmental fate of surfactants.

## Consideration 1: possible biodegradation scenarios

**Fig. 2:**
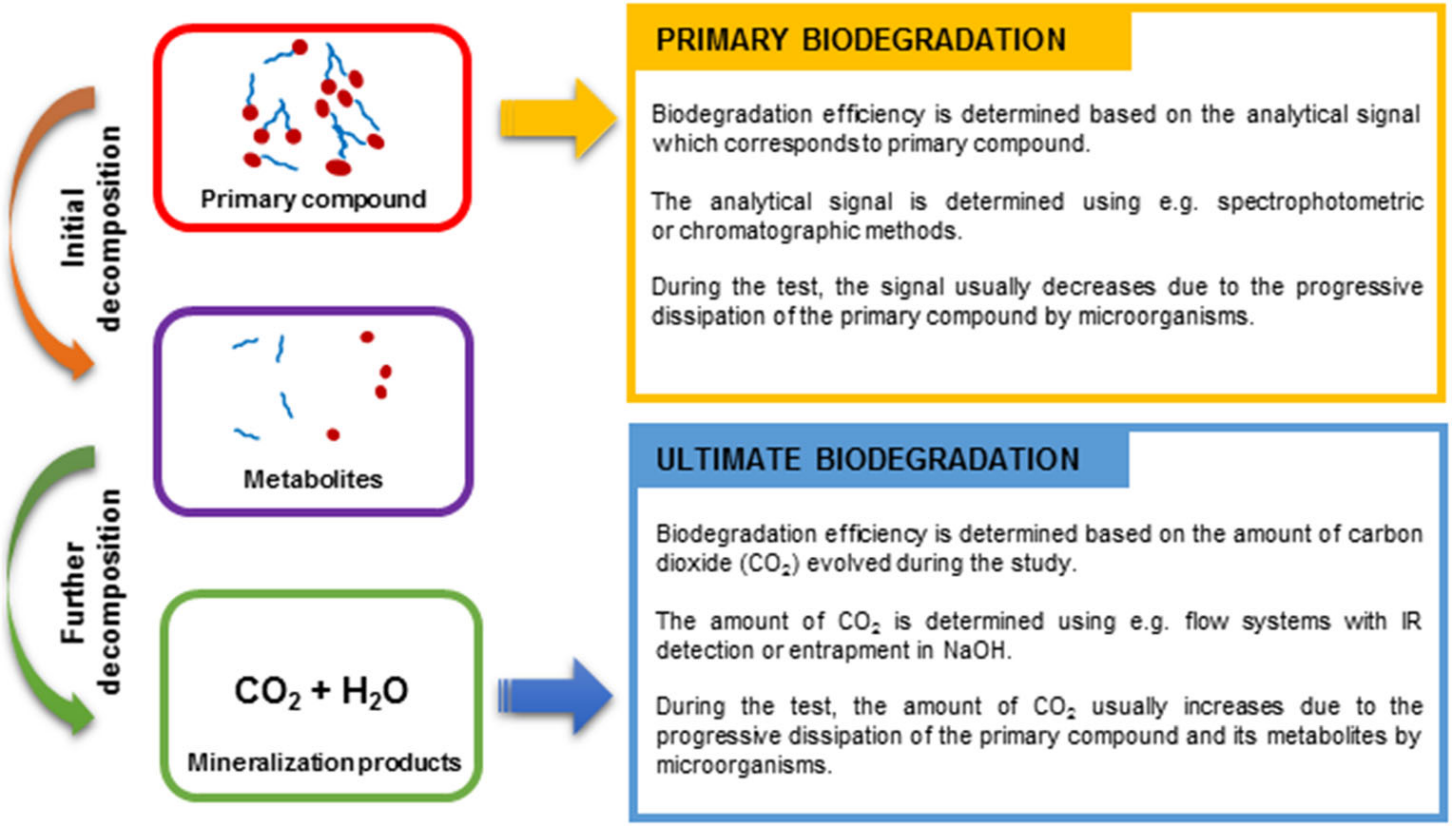
General overview of the tests systems employed for assessment of primary and ultimate biodegradation.

In order to properly evaluate the biodegradability of surfactants, it is necessary to understand the nature of biodegradation processes. As a general concept, “biodegradation” is the transformation of a given compound into a product characterized by a less complex chemical structure carried out by biological factors, mainly microorganisms (Neilson and Allard 2008). This definition can be further narrowed in order to distinguish two following terms: primary biodegradation, which corresponds to the dissipation of the parent compound, and ultimate biodegradation, which is associated with the complete mineralization of a given compound resulting in the formation of CO_2_, H_2_O, energy, and biomass. A conceptual scheme regarding the test systems which are used to investigate primary and ultimate biodegradation is presented in Fig. 2.

Test systems focused of the determination of primary biodegradation efficiency rely on the detection of the analytical signal attributed to the primary compound (in this case, the initial structure of the surfactant). The efficiency of primary biodegradation is calculated based on the decrease of this analytical signal. Detection is frequently carried out using a spectrophotometric approach; however, in cases when the surfactant occurs in the form of a mixture of homologues (e.g., Triton X-100) or when the analyte is present in a complex environmental matrix (e.g., soil), chromatographic methods are typically employed (usually HPLC, GC is also used although less frequently). The analytical procedures often require additional steps, e.g., derivatization.

Systems dedicated to the analysis of ultimate biodegradation investigate the efficiency of conversion of the primary compound to the most basic final metabolite—carbon dioxide. Assuming that microbial activity is not completely inhibited by the analyzed surfactant, the amount of evolved CO_2_ constantly increases during the study period and the efficiency of the process is calculated based on its final amount. The analytical procedure requires gas-tight conditions, and the determination is carried out by allowing direct transfer of the analyte to the detector (flow systems) or its entrapment in NaOH and the use of, e.g., calibrated pressure-sensitive sensors.

It should be emphasized that while both terms refer to processes of biological decomposition, their respective informational value is fundamentally different in terms of the possible fate of surfactants in the environment.

Primary biodegradation or biotransformation is, at best, an estimation of the susceptibility of a selected chemical compound to undergo a structural transformation due to the enzymatic activity of microorganisms (Sydow et al. 2015). Without supplementary assays (e.g., determination of possible metabolites), it does not provide any relevant data regarding the actual environmental impact of such a transformation (e.g., the stability and toxicity of the formed product). These limitations are often ignored during the interpretation of data, which leads to overestimation of biodegradation and false conclusions.

In this regard, the assessment of ultimate biodegradation or biomineralization is a more credible source of data for analysis of environmental impact, as it assumes a complete removal of both the initial compound and all subsequent metabolites (OECD, 301 1992). Furthermore, the measurement of carbon dioxide evolution directly reflects the activity of microorganisms and may hence indicate the occurrence of any toxic effects. While experimental setups based on ultimate biodegradation require adequate preparation to warrant acceptable accuracy of data, they contribute to a more comprehensive elucidation of environmental hazards.

The common mental shortcut, associated with the use of the general term “biodegradation” as an abbreviation which actually refers to experiments focused on primary biodegradation, is the main source of conceptual bias in numerous scientific reports.

## Consideration 2: depletion of surfactants not associated with biodegradation

Due to the common prevalence of surfactants in the environment, ease of use and low analysis time have become important traits of methods used for their monitoring (Shaharom et al. 2018). As a result, procedures which allow for accurate and rapid measurement of surfactant concentrations have attracted much popularity. Nevertheless, it should be noted that excessive simplification of analysis protocols may also contribute to a flawed interpretation. Numerous methods used for detection and quantification of surfactants (e.g., with the use of chromatography or spectrophotometry) rely on the differentiation between the analytical signals measured for initial and final samples in order to evaluate the depletion of surfactants. Therefore, the second major conceptual pitfall is associated with the fact that the determined decrease of surfactant content in a system comprising microorganisms is often unconditionally attributed to its biodegradation.

In order to properly identify the cause for the observed surfactant depletion, the entire analytical protocol has to be taken into consideration (Fig. 1). It has to be emphasized that, due to their amphiphilic structure, surfactants exhibit a strong tendency to adsorb to virtually any given surface (Langevin 2014). While in most cases desorption processes allow for satisfactory recovery of surfactants into the bulk phase, the potential of an irreversible loss of a fraction of surfactants should always be considered. This possibility is of particular importance in the case of relatively simple experimental designs (e.g., flasks studies), which only measure the reduction of the native compound but do not account for individual mechanisms that are able to reduce the effective concentration of surfactants in the test setup. A momentary depletion may be caused by the sorption of surfactants to the walls of the vessel used for experiments, regardless of the material (glass, steel, ceramics) (Timmer and Droge 2017). Surfactants may also be strongly bound to the interface of a liquid-liquid system (Langevin 2014), which also leads to the decrease of their content during extraction steps. The obvious loss of the analyte resulting from any employed filtration steps should also be accounted for in the final calculations. However, interactions between surfactants and microbial cells are perhaps the most often disregarded reason for analytical bias in biodegradation studies.

## Consideration 3: sorption to biomass

The fact that surfactants do affect cellular membranes is well known and commonly accepted. Several comprehensive reports confirmed that surfactant-mediated destabilization and subsequent disruption of cell membranes due to the formation of mixed surfactant-phospholipid micelles is one of the major mechanisms associated with the toxicity of surfactants (Carmona-Ribeiro and de Melo Carrasco 2013; Borkowski et al. 2016; Liu et al. 2016). Taking into account that incorporation of surfactants into the phospholipid bilayer cannot be considered a scientific novelty to date, it is surprising that this phenomenon is often neglected in case of biodegradation trials.

**Fig. 3:**
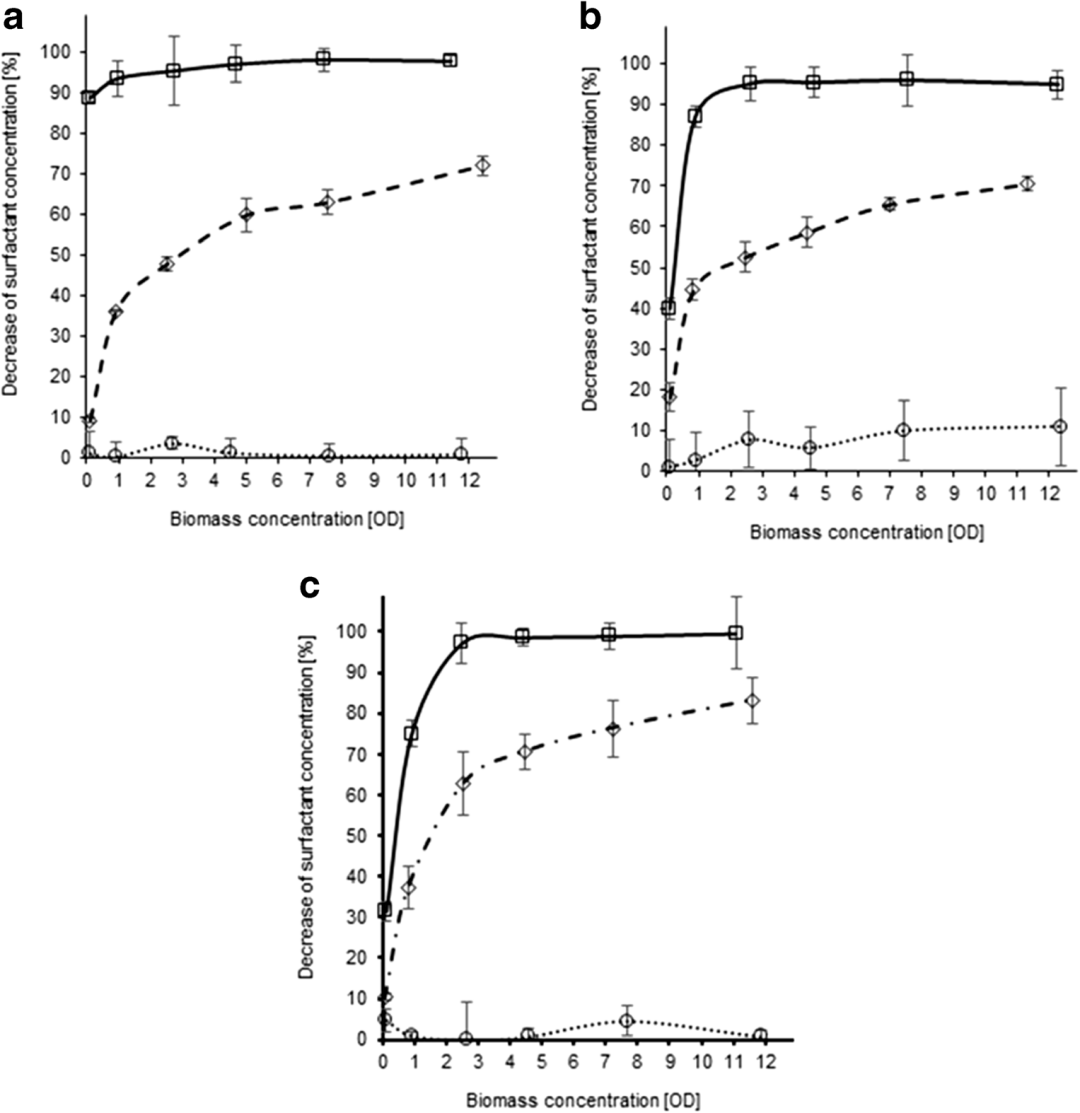
Sorption of surfactants (square—cationic, diamond—anionic, circle—non-ionic; initial concentration of 10 mg/L) after short-term exposure (1 min) to monoculture biomass (**a***P. putida*, **b***B. cereus*, **c***S. cerevisiae*) at different concentrations (expressed as OD, ranging from 0.1 to 12)

The majority of analytical procedures require the separation of cells from the medium (e.g., by centrifugation), and subsequent determination procedures are usually focused solely on the supernatant. Questions addressing how much of the original surfactant content is lost due to sorption on the biomass pellet and whether this amount is of any relevance or if it may be safely neglected often remain unanswered. In order to underline the importance of this issue, results of an original study focusing on the sorption of surfactants onto biomass are presented below (Fig. 3).

The objective of this investigation was to determine surfactant sorption (constant concentration of 10 mg/L) to different types of biomass considering the following main groups of microorganisms: *Bacillus cereus* (Gram-positive bacteria), *Pseudomonas putida* (Gram-negative bacteria), *Saccharomyces cerevisiae* (yeast), and activated sludge (most commonly tested complex mixed culture). The relevant values of biomass (g/L) corresponding to OD values presented for each studied microbial system in Fig. 3 are given in Table S1 in the Online Resource. Furthermore, calibration curves for the analytical procedures were presented in Figures S1–S3, whereas the reagents used for the experiments and calculations of LOD, LOQ, and RSD were presented in Tables S2–S9 in the Online Resource. The decrease of the analytical signal associated with the surfactant content was measured 1 min after contact of the biomass and the surfactant (Fig. 2). Analysis of the obtained results revealed three important implications. Firstly, sorption of surfactants depends on their type, i.e., the chemical structure. The following order which describes the susceptibility of surfactants to sorption on biomass may be established: cationic surfactants > anionic surfactants >> non-ionic surfactants. Sorption of cationic surfactants reached the highest values at the lowest corresponding OD (or g/L in case of activated sludge), regardless of the tested systems. Anionic surfactants exhibited a moderate tendency for sorption on biomass, whereas sorption of non-ionic surfactants was marginal for the majority of the studied biomass types (Fig. 3).

Secondly, the extent of surfactant sorption differed with regard to the type of microorganisms used in the study. For example, at the lowest biomass concentration (OD = 0.1 or 0.05 g/L in case of activated sludge), the content of cationic surfactants was reduced by 80–90% in systems comprising *P. putida* and activated sludge biomass, whereas in case of *B. cereus* and yeast cells, the reduction amounted to 30–40%. It should also be emphasized that notable sorption of non-ionic surfactants was observed only in case of activated sludge biomass (15–90% depending on the OD value).

Finally, the sorption of surfactants increased with the increase of the amount of biomass in the system. This effect was most apparent for anionic surfactants, as a gradual intensification of sorption was established for all studied systems (usually from approx. 10% to values > 70%). Even after the first increase of biomass concentration, the sorption was increased by a factor of two to five, depending on the type of biomass used for the experiment.

The notably higher affinity of cationic surfactants towards biomass may be explained by the structure of the cell envelope. The surfaces of microbial cells are characterized by an overall negative charge, due to the presence of teichoic acids in cell walls of Gram-positive bacteria, lipopolysaccharides in cell walls of Gram-negative bacteria (Van Loosdrecht et al. 1987; Magnusson and Johansson 1977), and phosphate groups in cell walls of yeast (Wilcocks and Smart 1995), which facilitate the transport of cationic surfactants from the bulk phase into the direct vicinity of the cells via electrostatic attraction (García et al. 2006). At this point, interactions of specific surfactant types with the phospholipid bilayer, which correspond to the initial stages of membrane solubilization mechanism, become the predominant factor (Otzen 2017). The surfactant monomer penetrates through the extracellular compounds and inserts into the bilayer (Jones 1999). Since both cationic and anionic surfactants are usually characterized by a strictly defined amphiphilic structure (the charged hydrophilic “head” may be easily distinguished from the hydrophobic “tail”), which closely resembles that of a phospholipid unit, they may be preferentially incorporated into the lipid section of the bilayer (Markiewicz et al. 2015), which corresponds well with their high sorption. In contrast, the structure of non-ionic surfactants deviates from the classic head-tail approach, which may render their embedding into the membrane hindered. Additionally, there are reports regarding membrane domains based on sphingolipids and cholesterol, which limit the membrane solubility processes by Triton X-100 (Koynova and Tenchov 2001). This may also explain the low sorption of the non-ionic surfactants to the biomass of studied monocultures. The fact that highest sorption was observed in case of experiments involving activated sludge biomass may be attributed to the formation of flocs (Baena-Nogueras et al. 2013). This peculiar matrix, which consists of cells incorporated in extracellular polymeric substances (EPS), is characterized by a notably higher surface area compared to traditional biofilms or planktonic cells. Furthermore, the presence of EPS may contribute to additional interactions, which would explain the higher sorption of non-ionic surfactants. Consequently, increased amount of biomass corresponds to enhanced sorption of the surfactants.

## Recommendations for accurate biodegradation assessment

**Fig. 4:**
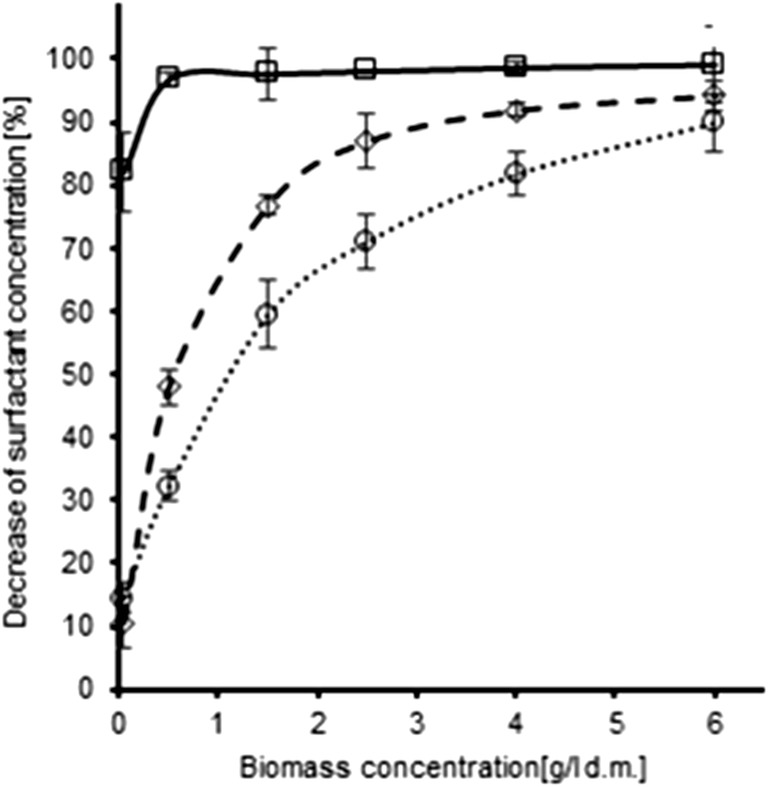
Sorption of surfactants (square—□ – cationic, diamond—◊ - anionic, circle—○ – non-ionic; initial concentration of 10 mg/Ll) after short- term exposure (< 1 min) to activated sludge biomass at different concentrations (expressed as g of dry matter per literlitre, ranging from 0.05 to 6).

The previously described phenomena may directly result in the overestimation of surfactant biodegradation efficiency. The overview of analytical protocols used for the determination of surfactant biodegradability in recently published scientific reports (presented in Table 1) revealed that in numerous cases, it is impossible to clearly distinguish whether the reduction of surfactant concentration occurred due to biodegradation or sorption. This particularly applies to studies in which the quantification of the surfactant content was based solely on the decrease of the analytical signal of the native compound. It should also be emphasized that the majority of the reviewed studies was carried out with the use of activated sludge, which exhibited the highest sorption capacity of surfactants in our study. Based on the investigated reports, the following guidance was formulated which will allow to obtain accurate and reliable biodegradation results (Fig. 4).

**Table 1:** Overview of analytical protocols used for the determination of surfactant biodegradability

Type of surfactant	Microorganism(s)	Experimental setup	Initial concentration	Removal efficiency	Analytical method	Comment	Reference
Cationic surfactant—cetylpyridinium chloride (CPC)	Activated sludge	Fed-batch bioreactor	0.5 mg/L 0.05 mg/L	90% (0.05 mg/L) and 66% (0.5 mg/L) after 42 days	HPLC	Biomass was included during the recovery of the surfactant; sorption of the surfactant was taken into account.	Nguyen and Oh (2019)
Cationic surfactant—cocamidopropyl betaine (CAPB)	Activated sludge as well as *Pseudomonas* sp. and *Rhizobium* sp. strains	Aerobic flask experiments	200 mg/L (activated sludge), 300 mg/L (strains)	63% mineralization after 10 days (activated sludge), 90% after 4 days (strains)	CO_2_ respirometry (activated sludge), dissolved organic carbon (strains)	Sorption of surfactants is not an issue in case of mineralization studies; however, it was not taken into account during the determination of dissolved organic carbon. Measurement of dissolved organic carbon (DOC) is not a selective method and the results may be influenced by cell metabolites.	Merkova et al. (2018)
Cationic gemini surfactants	Activated sludge	CO_2_ headspace biodegradation test	12 mg C/L	2% after 28 days	CO_2_ respirometry	Due to the employed methodology the obtained results are not influenced by sorption of surfactants on the biomass.	Kaczerewska et al. (2018)
Cationic surfactants—quaternary ammonium compounds (QACs)	Activated sludge + *Pseudomonas putida* (ATCC 12633) and *Aeromonas hydrophila* MFB03 immobilized on alginate beads	Aerobic flask experiments	200 mg/L	90% after 24 h	Colorimetric method with the use of bromothymol blue	Sorption of surfactants on biomass, biomass immobilized on alginate beads and biomass-free alginate beads was determined. Due to the employed analytical method it is unclear whether the surfactant was mineralized or biotransformed.	Bergero and Lucchesi (2018)
Cationic gemini surfactants	River water microorganisms	OECD 301D	3 mg/L	72–77% after 28 days	Biochemical oxygen demand measurement	Sorption of surfactants is not an issue in case of mineralization studies; however, no control samples were included in the test, which would allow to exclude the presence of organic carbon.	Xu et al. (2017)
Anionic surfactants—sodium dodecylbenzenesulfonate (SDBS), sodium dodecyl sulfate (SDS), sodium lauryl ether sulfate (SLES)	3 monocultures: *A. faecalis*, *E. cloacae*, and *S. marcescens*	Aerobic flask experiments	10 mg/L	20–90% for SDBS, 25–95% for SDS, and 15–50% for SLES after 6 days	Methylene blue active substances (MBAS)	No data regarding surfactant recovery from biomass, no data regarding initial inoculum, the achieved OD values exceed the expected OD based on the amount of introduced carbon source by approx. 4 times.	Fedeila et al. (2018)
Anionic surfactant—sodium lauryl ether sulfate (SLES)	Soil microbial community	Soil microcosms (1 kg of soil)	75–100 mg/kg of soil	100% after 28 days	Pressurized liquid extraction + methylene blue active substances (MBAS)	It can be assumed that the issues associated with sorption were taken into account during the pressurized liquid extraction. Metabolites of SLES were not determined; hence, it is unclear whether the surfactant was mineralized or biotransformed.	Barra Caracciolo et al. (2019)
Anionic surfactants—linear alkylbenzene sulfonates (LAS)	Microbial community in commercial laundry wastewater and domestic sewage	Expanded granular sludge bed (EGSB) reactor	4 and 16 mg/L	79% after 98 days (4 mg/L) and 60% after 95 days (16 mg/L)	No detection method was given	The manuscript was focused on microbial community dynamics, whereas the analytical procedures associated with surfactant detection were not properly described. It is not possible to determine whether the removal of LAS results from degradation or sorption.	Centurion et al. (2018)
Anionic surfactants—sodium lauryl sulfate (SLS) and sodium lauryl ether sulfate (SLES)	Activated sludge	Anaerobic membrane bioreactor (AnMBR)	Approx. 110 mg/L (influent)	35–65% (effluent)	Methylene blue active substances (MBAS)	No data regarding surfactant recovery from biomass.	Cheng et al. (2018)
Anionic surfactant—sodium dodecyl sulfate (SDS)	Bacteria isolated from sediment and wastewater samples	Aerobic flask experiments	Approx. 29 g	37–51% after 10 days	HPLC	Biomass was not included in the determination of surfactants. It is plausible that the depletion of the surfactant concentration occurred due to sorption and not biodegradation.	Adekanmbi and Usinola (2017)
Anionic surfactants—linear alkylbenzene sulfonates (LAS)	Microbial communities from four freshwater and marine sediments	OECD 308	10 mg/L	0–63% after 160 days (depending on the sediment type)	Pressurized liquid extraction + UPLC-ToF-MS	Mass balance was carried out, based on the determination of LAS in aqueous and particulate phases.	Corada-Fernández et al. (2018)
Anionic surfactants—linear alkylbenzene sulfonates (LAS)	Activated sludge	Aerobic bottle tests	50–250 mg/L	54–100% after 10 days	Biochemical oxygen demand + HPLC	Biomass was not included in the determination of surfactants. Since LAS removal was based on HPLC results, it is plausible that the depletion of the surfactant concentration occurred due to sorption and not biodegradation.	Katam et al. 2018
Anionic surfactant—sodium lauryl ether sulfate (SLES)	Activated sludge	Aerobic and anoxic bottle tests	500 mg/L	78% (aerobic) and 41% (anoxic) after 14 days	Dissolved organic carbon test	Biomass was not included in the determination of surfactants. It is plausible that the depletion of the surfactant concentration occurred due to sorption and not biodegradation.	Paulo et al. (2017)
Non-ionic surfactant—nonylphenol polyethoxylate	Activated sludge, anaerobically digested sludge	Anaerobic semi-continuous digesters	3 mg/L	90% after 52 days	GC/MS	Biomass was included during the recovery of the surfactant	Kara Murdoch et al. (2018)
Non-ionic (amphoteric) surfactants—alkyl amine oxides	Activated sludge	OECD 314A and OECD 303A tests	25 and 50 μg/L	76% after 4 days (OECD 314A test) 97% after 36 days (OECD 303A test)	Liquid scintillation counting and radiolabelled thin layer chromatography	Comprehensive analysis of C^14^-labelled surfactant depletion was conducted and mass balance was carried out. Biomass was included during the recovery of the surfactant; Sorption of the surfactant was taken into account.	McDonough et al. (2018)
Non-ionic surfactants—alkyl ethoxylates (AEOs), nonylphenol ethoxylates (NPEOs) and polypropylene glycols (PPGs)	Groundwater and soil microorganisms	Anaerobic microcosms	Working concentration of 350 mg/L (after combination with groundwater)	90–99% after 15–19 days (AEOs), 100% after 19 days (NPEOs), 68% after 50 days (PPGs)	LC-ToF-MS, GC-MS	Sorption of the surfactant on biomass was taken into account. Mass balance was carried out and potential metabolites were analyzed.	Heyob et al. (2017)
Non-ionic (amphoteric) surfactants—alkyl amine oxides	Sludge from anaerobic digester	OxiTop Closed bottle test	100 mg organic carbon/L	60% after 22 days	CO_2_ and CH_4_ respirometry	Due to the employed methodology the obtained results are not influenced by sorption of surfactants on the biomass.	Rios et al. (2017)
Non-ionic surfactants—ethoxylated fatty alcohols and ethoxylated fatty acid methyl esters	Activated sludge	Small activated sludge plant unit	10 mg/L	84–94% after 21 days	Bismuth-active substance (BiAS) test	Biomass was not included in the determination of surfactants. It is plausible that the depletion of the surfactant concentration occurred due to sorption and not biodegradation.	Szwach and Lukosek (2017)

**Fig. 5:**
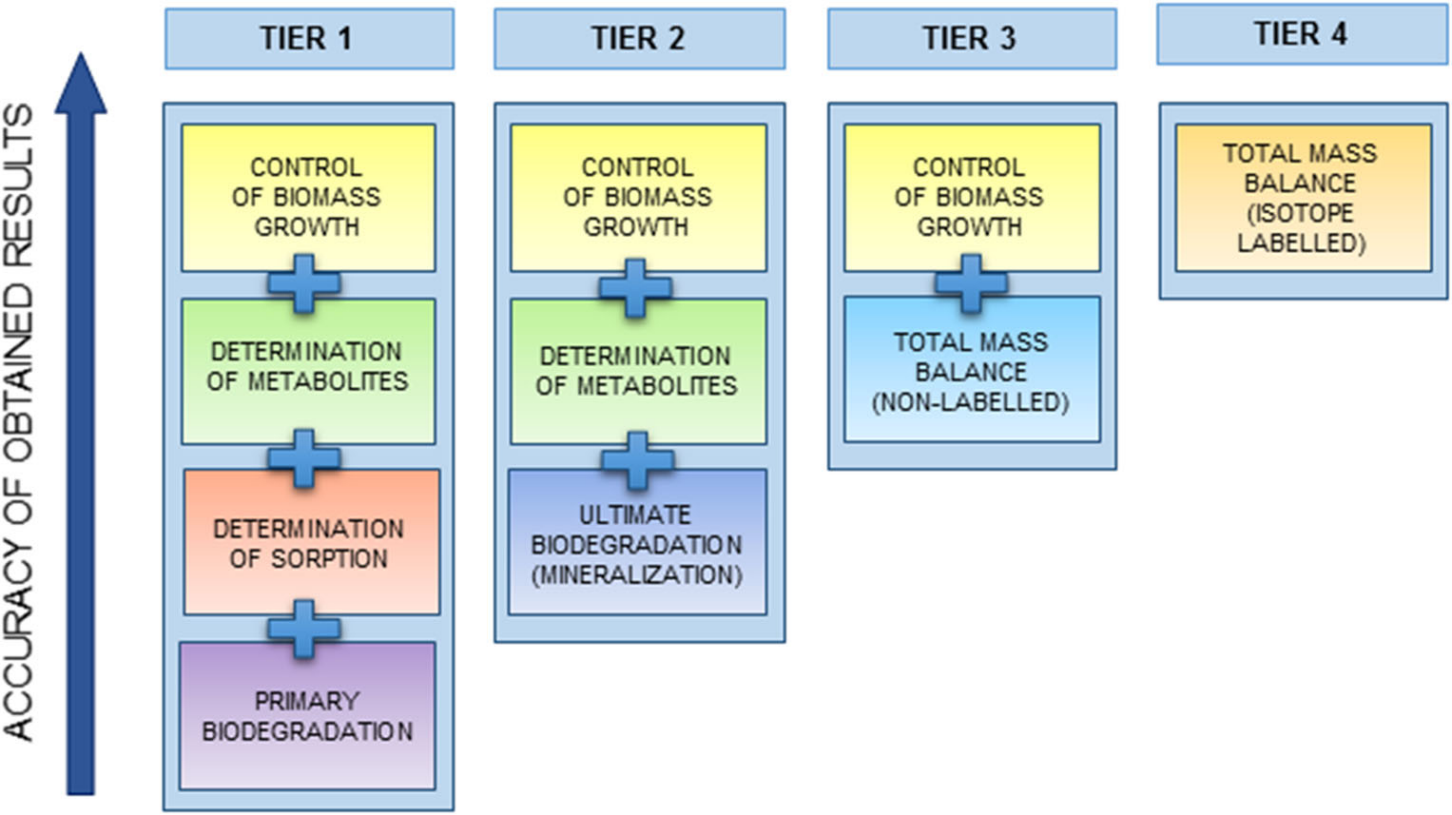
Tiered approach for increasing the accuracy of surfactant biodegradation test

Figure 5 presents recommended approaches for evaluating the biodegradation of surfactants divided into four tiers with a gradually increasing informational value regarding the environmental impact and reduced chances of bias. The bottom of tier 1 (primary biodegradation) is practically limited to the estimation of residual concentrations of the primary compound. When studies regarding sorption are included, the informational value of the approach is increased to the level of the bottom of tier 2 (ultimate biodegradation). At this level, it is possible to evaluate the amount of primary compound which was biodegraded and the residues with relatively high accuracy. To further elucidate the fate of the biodegraded surfactant, it is necessary to include an analysis of the metabolites formed during the process and to identify any stable and persistent structures. This step allows to reach the bottom of tier 3 (total mass balance). At this point, the studies should allow to establish the residual concentration of the surfactant, the decrease of surfactant content due to sorption (both abiotic and biotic), formation of stable metabolites due to transformation of initial surfactant structure, and dissipation of the surfactant. The “missing piece of the puzzle” is associated with the amount of surfactant which was used by the microorganisms as a source of carbon for anabolic processes. In consequence, it is advised to additionally monitor the biomass in order to ensure that decrease of the surfactant content correlates with cellular growth. This results in a highly accurate and credible evaluation, which is roughly at a similar level to tier 4—total mass balance using isotope-labelled compounds.

To summarize, in cases when studies are focused exclusively on primary biodegradation of surfactants, the implementation of surfactant sorption into the analytical protocol is a basic requirement which is necessary to exclude potential inconsistency of results. Moreover, in order to properly address the actual environmental impact of the tested compounds, such studies should include additional analyses, such as determination of potential metabolites (which especially applies to commonly used and commercially available surfactants) and evaluation of microbial growth (which indicates that the decrease of surfactant content may be attributed to the activity of microorganisms and allows to monitor potential toxic effects). Methods based on isotope-labelled compounds are characterized by the highest compliance with this approach as they also indicate the amount of studied compound which is incorporated into the biomass; however, their use is not always feasible due to high costs and the necessity to employ dedicated analytical apparatuses. Mass balance based on non-labelled compounds may also be carried out, assuming that the recovery of surfactants, determination of metabolites, and evaluation of carbon dioxide are included in the analytical protocol.

In conclusion, based on the list of reviewed publications as well as numerous earlier reports, it is evident that analytical approaches focused on the determination of surfactant biodegradation vary in terms of numerous practical aspects (ease of use, duration, selectivity, limits of detection and quantification, etc.). A major difference, which is not as obvious as can be expected, is associated with the susceptibility of such approaches to errors resulting from improper handling of biomass. In case of several publications reviewed in the framework of this study, it was not possible to establish whether the decrease of the analytical signal observed by the authors actually resulted from biodegradation of the surfactant. Without a comprehensive preparation of the experimental system, which includes the indication of possible sources of errors and development of appropriate counter-measures, the accuracy of the obtained results may be questionable.

We emphasize the necessity to consider the possibility of surfactant sorption onto microbial cells, which may result in significant detection errors as well as conceptual inconsistency. This particularly applies to systems which include ionic surfactants and activated sludge as sorption may account for 90% of the observed depletion of the surfactant. Coincidentally, such systems are most commonly applied in degradation studies, which further highlight the importance of this issue.

We propose a systematic approach in order to improve the credibility of the obtained results and limit the uncertainty which may result from sorption. Depending on the employed experimental setup, additional procedures such as determination of sorption to various elements of the test system, analysis of metabolites formed after the decomposition of the primary compound, and control of biomass growth may be required in order to verify that the decrease of surfactant concentration results from biodegradation processes.

Funding information

Financial support is given by the European Union’s Horizon 2020 research and innovation program under grant agreement no. 633962 for the project P4SB.

## Electronic supplementary material

ESM 1 (PDF 595 kb)
