# Supplementary material for: How to accurately assess surfactant biodegradation-impact of sorption on the validity of results
Source: Appl Microbiol Biotechnol. 2019 Nov 15;104(1):1–12. doi: 10.1007/s00253-019-10202-9 (PMC6942571; doi:10.1007/s00253-019-10202-9)
Supplement: Supplementary file 1 — (PDF 595 kb) [file 253_2019_10202_MOESM1_ESM.pdf]

Applied Microbiology and Biotechnology

How to accurately assess surfactant biodegradation - impact of sorption on the validity of results

Dorota Cierniak[1], Marta Woźniak-Karczewska[2], Anna Parus[2], Bogdan Wyrwas[1], Andreas P. Loibner[3], Hermann J. Heipieper[4], Łukasz Ławniczak[2],*, Łukasz Chrzanowski[2,4]

[1] Institute of Chemistry and Technical Electrochemistry, Poznan University of Technology, Bedrychowo 4, 60-965 Poznan, Poland

[2] Institute of Chemical Technology and Engineering, Poznan University of Technology, Pl. M. Skłodowskiej-Curie 2, 60-965 Poznan, Poland

[3] IFA-Tulln, BOKU-University of Natural Resources and Life Sciences, Vienna, 3430 Tulln, Austria

[4] Helmholtz Centre for Environmental Research – UFZ, Department of Environmental Biotechnology, Permoserstraße 15, D-04318 Leipzig, Germany

* Corresponding author: Łukasz Ławniczak

E-mail address: lukasz.lawniczak@put.poznan.pl

Telephone number: + 48 61 6653716

Evaluation of surfactant sorption

1. Cultivation of microorganisms

In order to determine the sorption capability of biomass, different species were used throughout the studies: *Pseudomonas putida* (Gram negative bacteria), *Bacillus cereus* (Gram positive bacteria) and *Saccharomyces cerevisiae* (yeast). The microorganisms were kindly provided by the Geomicrobiology Laboratory (Faculty of Geology, University of Warsaw). Additionally, the studies included the evaluation of sorption capability of activated sludge, which was collected between June and August 2018 from the main wastewater treatment plant in Kozieglowy near Poznan (Poland).

Due to the various substrate preferences, the pre-cultures for the experiments were set up differently in order to obtain a sufficient amount of biomass. Briefly, *S. cerevisiae* was cultivated using glucose (15 g/L) as the sole carbon source, according to the procedure provided by Behrens et al. (1978); *P. putida* was cultivated using a mineral medium supplemented with sodium succinate (15 g/L) as described by Hartmans et al. (1989); while *B. cereus* was cultivated using a TSB broth (Oxoid, UK). The pre-cultures (500 mL) were incubated at 30 °C for 72 h in sterile 2 L Erlenmeyer flasks which were placed in a rotary shaker (120 rpm). At the end of logarithmic phase, each cell suspension was centrifuged at 4,500 × g for 15 min at 4 °C and the isolated biomass was washed three times with a sterile NaCl solution (0.85% w/v). Subsequently, the bacterial cells were re-suspended in 30 mL of NaCl solution to obtain a cell dry weight [g/L] equal to approx. 0.05; 0,5; 1,5; 2,5; 4; 6. The activated sludge was used immediately after sampling and washed as described above with additional sieving through a 400 μm sieve to remove solid particles. All samples, abiotic (NaCl solution with surfactant) and biotic (NaCl solution with biomass, without surfactant) controls were prepared in five replicates and incubated at 25°C with constant shaking (60 rpm). The samples for analyses were collected every 12 h over two days.

2. Experimental set-up

The microbial biomass prepared as described above was introduced into 30 ml of 0.85% NaCl solutions in an amount which allowed to obtain the assumed OD values: 0.1; 1; 3; 5; 8 and 12

(Table S1). Then an appropriate surfactant (hexadecyltrimethylammonium chloride, sodium dodecylbenzenesulfonate or Triton X-100) was added to the solutions in an amount which allowed to reach the concentration of 10 mg/L.

Table S1. Biomass of studied monocultures used to obtain the assumed OD values

Type of surfactant	*P. putida*		*B. cereus*		*S. cerevisiae*	
	**Biomass [g/l d.m.]**	**Optical density**	**Biomass [g/l d.m.]**	**Optical density**	**Biomass [g/l d.m.]**	**Optical density**
**Cationic**	4,688	11,435	7,248	12,285	5,878	11,090
	3,053	7,446	4,458	7,556	3,757	7,088
	1,929	4,704	2,739	4,642	2,345	4,425
	1,130	2,755	1,548	2,624	1,307	2,466
	0,390	0,951	0,523	0,887	0,470	0,887
	0,040	0,098	0,057	0,096	0,051	0,095
**Anionic**	5,088	12,410	6,667	11,300	6,143	11,590
	3,106	7,576	4,148	7,030	3,823	7,214
	2,061	5,026	2,603	4,412	2,354	4,441
	1,038	2,532	1,438	2,437	1,344	2,536
	0,373	0,910	0,479	0,812	0,424	0,800
	0,036	0,089	0,053	0,090	0,044	0,083
**Non-ionic**	4,820	11,755	7,295	12,365	6,283	11,855
	3,129	7,632	4,401	7,460	4,067	7,674
	1,856	4,528	2,657	4,504	2,410	4,548
	1,099	2,680	1,534	2,600	1,402	2,646
	0,365	0,890	0,527	0,893	0,478	0,901
	0,039	0,095	0,056	0,096	0,053	0,100

Afterwards, the solutions were constantly stirred using a rotary shaker (60 rpm at 25°C). Samples (7 ml) were collected after the assumed time periods (1 min, 12 h, 24 h and 48 h) into 8 ml tubes. After centrifugation (4500 rpm for 15 min), 6 ml of the supernatant was introfuced into vials which contained 100 μl of formalin and subjeced to determination of residual surfactant content.

3. Analysis of residual surfactant content

3.1 Determination of cationic surfactant - hexadecyltrimethylammonium chloride - Disulphine Blue Active Substances (DBAS) method

Cationic surfactants were determined using the DBAS (Disulphine Blue Active Substances) method, which is based on the reaction of cationic surfactants with an anionic reagent - disulphine blue (Table S1). The formed ionic pair (surfactant - disulphine blue) was later extracted with chloroform and the absorbance of the solution was measured spectrophotometrically at 627 nm.

Table S2. Reagents used for the determination of cationic surfactant with the DBAS method

Reagent	Supplier	Purity	Preparation
hexadecyltrimethylammonium chloride	Sigma Aldrich, Germany	p.a.	0.1% aqueous solution
Chloroform	Avantor, Poland	p.a.	-
Buffer solution (pH = 5)	Avantor, Poland	p.a.	250 ml of an aqueous solution consisting of 35.61 g of anhydrous sodium acetate and 14.3 ml of concentrated glacial acetic acid was prepared.
Disulphine Blue	Avantor, Poland	p.a.	0.16 g of disulphine blue was dissolved in 20 ml of 10% (v/v) aqueous ethanol, then filled up to 250 ml with demineralized water.

Analytical procedure: 10 ml of demineralized water, 5 ml of sample (formalin-fixed supernatant), 2.5 ml of acetate buffer, 1 ml of disulphine blue solution and 5 ml of chloroform were introduced into a 100 ml separating funnel. The mixture was shaken for 3 minutes. After the separation of phases occurred (5 minutes), the lower chloroform phase was transferred into a glass cuvette (10

mm x 10 mm x 30 mm), then the absorbance was measured with a UV-Vis spectrometer Jasco V-530 (Japan).

The linearity of the method is in the range of 1÷75 μg in the sample, and the LOQ is 3 μg for the sample. The precision of the method (RSD) is equal to 4.19% (determined using actual samples with a content of approximately 20 μg). An exemplary calibration curve for the simplified DBAS method was presented in Fig. S1.

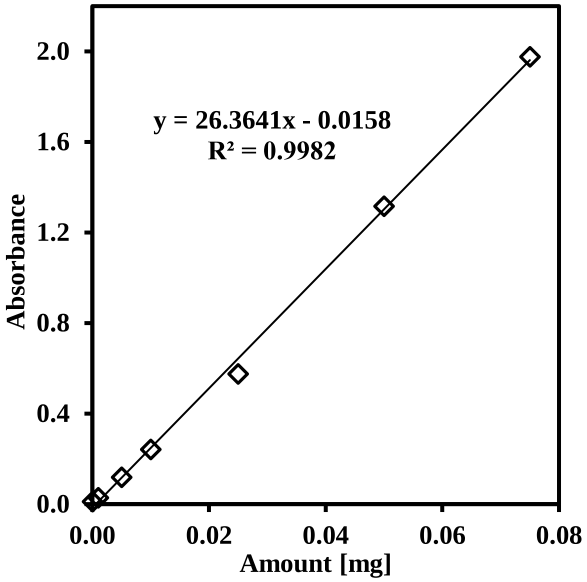

Fig. S1. An exemplary calibration curve for the DBAS method in the range of 1÷75 μg in a sample, the reference compound - cetyltrimethylammonium chloride.

3.2 Determination of anionic surfactant - sodium dodecylbenzenesulfonate - Methylene Blue Active Substances (MBAS) method

Anionic surfactants were determined using the MBAS (Methylene Blue Active Substances) method, which is based on the reaction of methylene blue, as the cationic reagent, and anionic surfactants (Table S3). The formed ionic pair (methylene blue – surfactant) was later extracted with chloroform and the absorbance of the solution was measured spectrophotometrically at 652 nm.

Table S3. Reagents used for the determination of anionic surfactant with the MBAS method

Reagent	Supplier	Purity	Preparation

Sodium dodecylbenzenesulfonate	Sigma, Aldrich, Germany	p.a.	0.1% aqueous solution
Chloroform	Avantor, Poland	p.a.	-
Methylene Blue (acidic aqueous solution)	Chempur, Poland	p.a.	1000 ml of an aqueous solution containing 0.35 g of methylene blue and 6.5 ml of conc. H_2SO_4 with a density of 1.84 g/ml in demineralized water.

Analytical procedure: 45 ml of demineralized water, 5 ml of sample (formalin-fixed supernatant), 5 ml of methylene blue and 10 ml of chloroform were introduced into a 100 ml separating funnel. The mixture was shaken for 3 minutes. After the separation of phases occurred (3 minutes), the lower chloroform phase was filtered (paper filters for qualitative analysis: Sartorius 3 m/N, basis weight 65 g/m^2) into a glass cuvette (10 mm x 10 mm x 30 mm). The absorbance was measured with a UV-Vis spectrometer Jasco V-530 (Japan).

The linearity of the method is in the range of 3÷100 μg in the sample, and the LOQ is 4 μg for the sample. The precision of the method (RSD) is equal to 7.86% (determined using actual samples with a content of approximately 20 μg). An exemplary calibration curve for the simplified MBAS method was presented in Fig. S2.

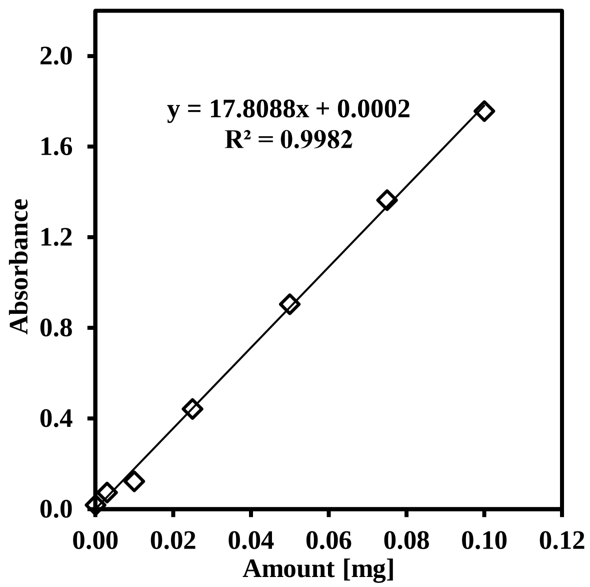

Fig. S2. An exemplary calibration curve for the simplified MBAS method in the range of 3÷100 μg in a sample, the SDBS standard

3.3 Determination of non-ionic surfactant – Triton X-100 - BiAS-thio method

Non-ionic surfactants were determined using the BiAS-thio method (Table S4).

Table S4. Reagents used for the determination of anionic surfactant with the BiAS-thio method

Reagent	Supplier	Purity	Preparation
Triton X-100	Sigma Aldrich, Germany	p.a.	0.1% aqueous solution
Acetic acid 99.5-99.9%	Avantor, Poland	p.a.	-
Dragendorff reagent with a reducing agent	Avantor, Poland	p.a.	1000 ml of an aqueous solution consisting of 1.2 g of basic bismuth nitrate (III), 150 ml of glacial acetic acid, 100 g of $BaCl_2 \cdot 2H_2O$, 50 g of potassium iodide and 5 g

			of anhydrous sodium phosphate (NaH_2PO_2) in demineralized water was prepared. After complete dissolution of the reagents and filling the flask with water to the mark, the solution was filtered through a medium filtering paper.
Dissolving-complexing solution (15% thiourea in 1 M HNO_3)	Avantor, Poland	p.a.	100 ml of an aqueous solution composed of 15 g of thiourea and 6.9 ml of conc. nitric acid (65%) in demineralized water were prepared.

Analytical procedure: 1 ml of Dragendorff reagent was added to a 8 ml centrifuge tube (PP), followed by 2 ml of the sample (formalin fixed supernatant). Then, after precipitation of the oxyethylates (2 min), the content of the tube was centrifuged (5 min, 12000 rpm) using a high-speed laboratory centrifuge MPW 352 (Poland). After discarding the liquid, the precipitate remaining in the tube was washed with glacial acetic acid (3 x 1 ml) to remove the residue of the Dragendorff reagent. Then, 2 ml of dissolving-complexing solution was added to the isolated and previously washed precipitate. The solution was introduced into a glass cuvette (10 mm x 10 mm x 30 mm) and the absorbance was measured with a UV-Vis spectrophotometer Jasco V-530 (Japan).

The linearity of the method is in the range of 2÷100 μg in the sample, and the LOQ is 3 μg for the sample. The precision of the method (RSD) is equal to 9.25% (determined using actual samples with a content of approximately 20 μg). An exemplary calibration curve for the simplified BiAS-thio method was presented in Fig. S3.

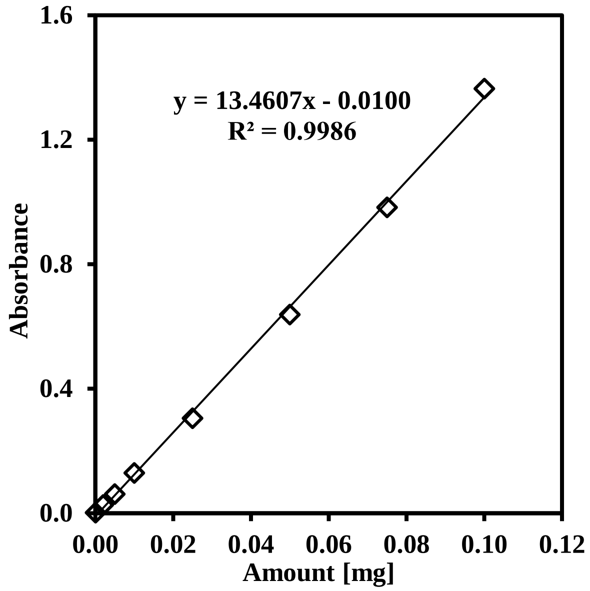

Fig. S3. An exemplary calibration curve for the simplified BiAS-thio method in the range of 2÷100 μg in a sample, TX-100 standard

3.4 Determination of limits of detection (LOD) and quantification (LOQ) as well as precision (RSD) of the analytical methods

The limits of detection (LOD) and quantification (LOQ) presented in Tables S5 and S6 for DBAS and MBAS methods, respectively, were determined using blank samples based on the following equations:

$$LOD = x_m + 3*SD$$

$$LOQ = 3*LOD$$

where: x_m – mean value [μg in sample]

SD – standard deviation [μg in the sample]

Table S5. Limit of detection and quantification of the DBAS method, determined for the blank samples

No. Sample	Absorption	Content

		[μg in the sample]
1	0.0090	0.910
2	0.0102	0.918
3	0.0082	0.937
4	0.0104	0.941
5	0.0084	0.986
6	0.0089	0.990
7	0.0103	0.994
mean value x_m		0.954
standard deviation SD		0.036
**LOD**		**1.06**
**LOQ**		**3.18**

Table S6. Limit of detection and quantification of the MBAS method, determined for the blank samples

**No. Sample**	**Absorption**	**Content [μg in the sample]**
1	0.0177	0.983
2	0.0190	1.055
3	0.0201	1.116
4	0.0204	1.134
5	0.0214	1.190
6	0.0245	1.364
7	0.0253	1.409
mean value x_m		**1.096**
standard deviation SD		**0.079**
**LOD**		**1.33**
**LOQ**		**4.00**

The precision of the determinations (RSD - relative standard deviation) was established by repeating the analysis of a given sample five times (sorption on yeast at OD = 0.1) with a content of approx. 20 μg of analyte in the sample (corresponding to approx. 4 mg/l) and the parameter was calculated based on the equation:

$$RSD = \frac{SD}{x_m} * 100\% \ [\%]$$

where: x_m – mean value [μg in sample]

SD – standard deviation [μg in the sample]

The RSD values for the respective methods (DBAS, MBAS and BiAS-Thio) were presented in Tables S7-S9.

Table S7. Calculation of RSD for the DBAS method

**No. sample**	**Content [μg in the sample]**	**concentration [mg/l]**
1	19.67	4.01
2	20.19	4.12
3	20.94	4.27
4	21.14	4.31
5	21.90	4.47
mean value x_m	20.77	4.24
standard deviation SD	0.86	0.18
**RSD**	**4.16**	**4.19**

Table S8. Calculation of RSD for the MBAS method

**No. sample**	**Content [μg in the sample]**	**concentration [mg/l]**

1	18.99	3.88
2	19.05	3.89
3	16.79	3.43
4	20.72	4.23
5	20.20	4.12
mean value x_m	19.15	3.91
standard deviation SD	1.51	0.31
**RSD**	**7.91**	**7.86**

Table S9. Calculation of RSD for the BiAS-thio method

No. sample	content [μg in the sample]	concentration [mg/l]
1	25.09	12.80
2	21.65	11.05
3	19.65	10.03
4	22.85	11.66
5	21.11	10.77
mean value x_m	22.07	11.26
standard deviation SD	2.04	1.04
**RSD [%]**	**9.25**	**9.24**

References

Behrens U, Weißbrodt E, Lehmann W (1978) Zur kinetik der Citronensa ürebildung bei *Candida lipolytica*. Z Allg Mikrobiol 18:549–558.

Hartmans S, Smits JP, van der Werf MJ, Volkering F, de Bont JAM (1989) Metabolism of styrene oxide and 2-phenylethanol in the styrene-degrading *Xanthobacter* strain 124X. Appl Environ Microbiol 55:2850-2855

Wyrwas B, Zgoła-Grześkowiak A (2015) A new iodobismuthate method with a low volume filtration device as a new tool for the determination of microgram oxyethylate amounts. Tenside Surfact Det 52:213-218

Kruszelnicka I, Ginter-Kramarczyk D, Wyrwas B, Idkowiak J (2019) Evaluation of surfactant removal efficiency in selected domestic wastewater treatment plants in Poland. J Environ Health Sci Eng
